# Generating *In Vivo* Cloning Vectors for Parallel Cloning of Large Gene Clusters by Homologous Recombination

**DOI:** 10.1371/journal.pone.0079979

**Published:** 2013-11-11

**Authors:** Jeongmin Lee, Eugene Rha, Soo-Jin Yeom, Dae-Hee Lee, Eui-Sung Choi, Seung-Goo Lee

**Affiliations:** 1 Biochemicals and Synthetic Biology Research Center, KRIBB, Yuseong-gu, Daejeon, Korea; 2 Biosystems & Bioengineering, University of Science & Technology, Yuseong-gu, Daejeon, Korea; Florida International University, United States of America

## Abstract

A robust method for the *in vivo* cloning of large gene clusters was developed based on homologous recombination (HR), requiring only the transformation of PCR products into *Escherichia coli* cells harboring a receiver plasmid. Positive clones were selected by an acquired antibiotic resistance, which was activated by the recruitment of a short ribosome-binding site plus start codon sequence from the PCR products to the upstream position of a silent antibiotic resistance gene in receiver plasmids. This selection was highly stringent and thus the cloning efficiency of the GFPuv gene (size: 0.7 kb) was comparable to that of the conventional restriction-ligation method, reaching up to 4.3 × 10^4^ positive clones per μg of DNA. When we attempted parallel cloning of GFPuv fusion genes (size: 2.0 kb) and carotenoid biosynthesis pathway clusters (sizes: 4 kb, 6 kb, and 10 kb), the cloning efficiency was similarly high regardless of the DNA size, demonstrating that this would be useful for the cloning of large DNA sequences carrying multiple open reading frames. However, restriction analyses of the obtained plasmids showed that the selected cells may contain significant amounts of receiver plasmids without the inserts. To minimize the amount of empty plasmid in the positive selections, the *sacB* gene encoding a levansucrase was introduced as a counter selection marker in receiver plasmid as it converts sucrose to a toxic levan in the *E. coli* cells. Consequently, this method yielded completely homogeneous plasmids containing the inserts via the direct transformation of PCR products into *E. coli* cells.

## Introduction

Modern biotechnology requires a robust method for rapid cloning and expression of large gene clusters, occasionally necessitating the parallel cloning of various DNA fragments [1,2]. Furthermore, the emergence of high throughput biotechnology and synthetic biology also accentuates the necessity of rapid cloning of large synthetic DNAs [3,4]. However, the conventional methods using restriction enzymes require extensive restriction and modification reactions that are highly dependent on the DNA sequences of the target genes, making this method time consuming and less-convenient for the cloning of large gene clusters [1]. 

Homologous recombination (HR) is a type of genetic recombination in which nucleotide sequences are exchanged between two identical sequences of DNA [5-7]. The HR reaction uses the high activity recombinase such as RecET [5,6,8,9] or Redαβ [7,10,11], and has been applied successfully as gene knock-out and replacement tools for chromosomes [5,10,11] and in BAC engineering in *Escherichia coli* [12,13]. The HR method has also been used for connecting linearized plasmids and PCR products in *E. coli* [14,15], demonstrating its particular advantage for the cloning of large genes [5,6], that are difficult to handle with conventional cloning technologies. While this method simplifies the cloning process by avoiding the ligation reaction of DNA fragments and giving more freedom for cloning, it still requires the steps of linear plasmid preparation either by PCR or by the linearization of an intact plasmid [5,6]. This may lead to many false-positives from incomplete linearization and /or the re-ligation of the linear plasmids [16-18]. 

**Figure 1:**
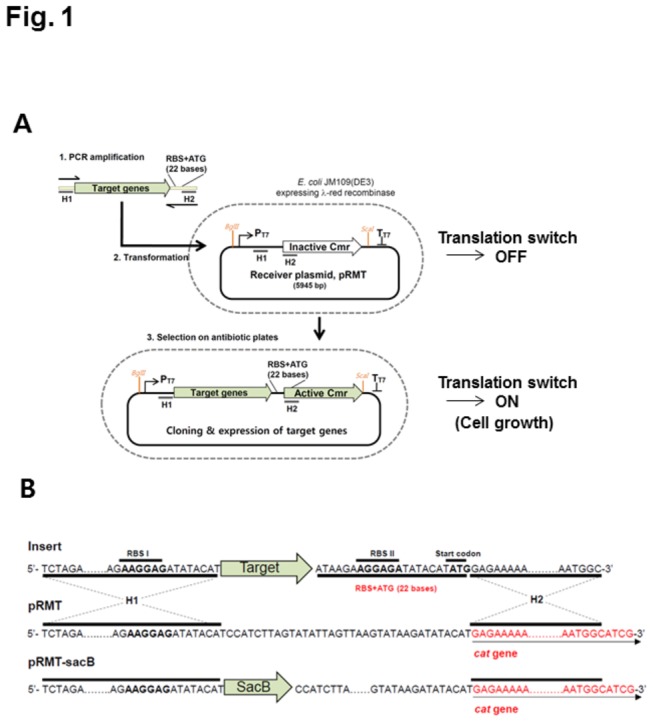
Strategy for *in* *vivo* cloning by the recruitment of the translation initiation sequence for antibiotic marker expression. (A) The receiver plasmid, pRMT, contains a silent chloramphenicol resistance gene (Cmr) that is activated by homologous recombination with the target DNA in *E. coli* cells. The DNA insert is comprised of 5'-homologous sequences (H1), target gene sequences, the RBS plus ATG for activation of the silent selection marker, and 3'-homologous sequences (H2). F and R indicate primers with specific sequences for the amplification of the target DNA. P_T7_ and T_T7_ represent the T7 promoter and T7 terminator, respectively. (B) Shown are the sequences of the homologous region used to prepare the inserts with the target gene and the region for homologous recombination with the silent chloramphenicol resistance gene in receiver plasmid, pRMT and pRMT-sacB.

Accordingly, in this study we propose a HR cloning system that recombines a PCR product into a receiver plasmid in *E. coli* cells without the need for any restriction or ligation steps. The growth on the antibiotic plate is activated by the recruitment of a ribosome binding site plus the start codon (RBS+ATG, 22 bp) from a PCR product to the upstream site of a silent antibiotic resistance gene in the receiver plasmid (Figure 1A). The RBS is an RNA region 6-7 nucleotides upstream of the start codon in prokaryotes that is bound by the ribosome when initiating the translation process. Therefore, the transformation of PCR products with this 22 base tag and the subsequent selection on and antibiotic plate may led to the rapid cloning of large genetic clusters in this study.

## Materials and Methods

### Materials

*E. coli* JM109 (DE3) was purchased from Promega (Madison, WI, USA). Arabinose for inducing lambda-Red recombinase and sucrose for the counter-selection using levansucrase were obtained from Sigma-Aldrich Co. (St Louis, MO, USA). Ampicillin, chloramphenicol, kanamycin, streptomycin, and tetracycline were purchased from Duchefa (Haarlem, The Netherlands). All restriction enzymes and ligases were purchased from Roche (Indianapolis, IN, USA) or Takara (Otsu, Shiga, Japan). DNA dephosphorylation was performed using shrimp alkaline phosphatase (Roche, Indianapolis, IN, USA). The DNA was amplified using Pyrobest (Takara, Otsu, Shiga, Japan), which is a *pfu* polymerase, while the large DNAs (>10kb) were amplified using KOD-FX polymerase (Toyobo, Osaka, Japan). Qiagen kits were used to purify the PCR products and for plasmid isolation. Oligonucleotides were purchased from Bioneer (Daejeon, Korea). All bacterial strains and primers used in this study are respectively listed in Table 1 and 2.

**Table 1:** Bacterial strains and plasmids used in this study.

**Strains or plasmid**	**Description**	**Source or reference**
***E. coli* strain**		
JM109(DE3)	endA1, recA1, gyrA96, *thi,* hsdR17, (rk-, mk+), relA1, supE44, λ-, Δ(*lac-*proAB), [F´, *tra*D36, proAB, *lac*IqZΔM15], lDE3	Promega
**Plasmid**		
pKD46	P_araB_αβγ, Amp^r^	[10]
pRMT	RMT cassette,pBR322 ori, Kmr	This Study
pRMT-sacB	RMT cassette, *sac*B, pBR322 ori, Kmr	This Study
pSTV28	pACYC ori, Cmr	Takara
pET-27b	P_T7_, pBR322 ori, Km^r^	Novagen
pKO3	*sac*B	[19]
pMGFP	MBP- GFP	[20]
pTrcAKpGalAZpTrcEBIY	*crt*EBIY	GenBank # GQ 149341
	*crt*W	GenBank# BAE47466
	*crt*Z	GeneBank# BAE47466
pSSN12Didi	*mavk*1,*mvak*D, *mvak*2, *idi*	[24]

**Table 2:** List of oligonucleotides used in this study.

**Oligo-nucleotide**	**Sequences (5’-3’)**
**For construction of pRMT and pRMT-sacB vector**	
1-1F	CCATCTTAGTATATTAGTTAAGTATAAGATATACATGAGAAAAAAATCAC
cat-R	TTACGCCCCGCCCTGCCACTCATCGC
rec-catF	TCTAGAAATAATTTTGTTTAACTTTAAGAAGGAGATATACATCCATCTTAGTATATTAGTTAAG
rec-catR	GTGGTGGTGGTGGTGGTGCTCGACATCCTCGGGGTCTTCCGGTTATTACGCCCCGCCCTGCCACTCATCGC
sacB-F	gaaataattttgtttaactttaagaaggagatatacatatgaacatcaaaaagtttgcaaaacaag
sacB-R	ctcatgtatatcttatacttaactaatatactaagatggttatttgttaactgttaattgtccttgttcaag
RMT-R2	cttgaacaaggacaattaacagttaacaaataaccatcttagtatattagttaagtataagatatacatgag
**For insert preparation**	
H1-GFPuvF	TCTAGAAATAATTTTGTTTAACTTTAAGAAGGAGATATACATATGAGTAAAGGAGAAGAACTTTTCACTGG
H2-GFPuvR	GCCATTGGGATATATCAACGGTGGTATATCCAGTGATTTTTTTCTC **CAT**ATGTATA**TCTCCT**TCTTATTTATTTG TAGAGCTCATCCATGCC
H1-crtEF	TCTAGAAATAATTTTGTTTAACTTTAAGAAGGAGATATACATATGAACAGTCCCTCTACCACTTTATTGC
H2-crtER	GCCATTGGGATATATCAACGGTGGTATATCCAGTGATTTTTTTCTC **CAT**ATGTATA**TCTCCT**TCTTATTCACGCCCATTTGAGTGCTG
H1-crtWF	TCTAGAAATAATTTTGTTTAACTTTAAGAAGGAGATATACATATGATTGCAGGAAATCAAATTTTAGGA
H2-idiR	gccattgggatatatcaacggtggtatatccagtgatttttttctc **cat**atgtata**tctcct**tcttatttatttaagctgggtaaatgcagataatc
H1-malEF	TCTAGAAATAATTTTGTTTAACTTTAAGAAGGAGATATACATATGAAAATCGAAGAAGGTAAACTGG
H2-malER	GCCATTGGGATATATCAACGGTGGTATATCCAGTGATTTTTTTCTC **CAT**ATGTATA**TCTCCT**TCTTATTTACTTGTACAGCTCGTCCATGC

Underlined H1 and H2 indicate the sequences for homologous recombination in pRMT plasmids. Bolded “CAT” indicates the start codon for the *cat* gene. Bolded and italic sequences are the RBS for the *cat* gene.

### Construction of HR-cloning plasmids pRMT and pRMT-sacB

The receiver plasmids, pRMT and pRMT-sacB were constructed using HR between two linear DNAs: the PCR products and linearized plasmids. The *E. coli* cells harbors the helper plasmid, pKD46, which encodes the *exo*, *bet*, and *gam* proteins of bacteriophage lambda and enables the efficient HR reaction [10]. 

The pET-27b(+) plasmid (Novagen, Germany) was linearized with the restriction enzyme *Bam*HI and dephosphorylated with the shrimp alkaline phosphatase. The silent *cat* gene, which encodes a chloramphenicol acetyltransferase missing the start codon, was prepared by PCR from pSTV28 (Takara Bio, Japan) using the primers 1-1F and cat-R (Table 2), and the resulting gene was subjected to a second round of PCR using the primers rec-catF and rec-catR to attach flanking homology sequences with the pET-27b(+) plasmid (Figure S1). The linearized plasmid and PCR product were then co-transformed via electroporation into *E. coli* JM109(DE3) cells containing pKD46, and grown in LB media with 100 μg/ml amplicillin and 50 mM arabinose at 30°C. The electroporation was conducted with 100 ng of the PCR product and 10 ng of the linearized pET-27b vector using the Gene Pulser Xcell System (Bio-Rad, Hercules, CA, USA). After electroporation, the cells were added with 1 ml of SOC medium, reactivated for 1 h at 37°C to express antibiotic resistance, and plated on LB agar medium containing 10 μg/ml of kanamycin. The positive clones were validated by colony PCR using a T7 promoter and T7 terminator primers. 

To construct the pRMT-sacB plasmid, the *sacB* gene, which encodes a levansucrase that synthesizes a harmful and toxic levan in *E. coli* cells, was amplified from pKO3 [19] using the primers sacB-F and sacB-R (Table 2, Figure S1). Then, the *cat* gene of pRMT was amplified using the RMT-R2 and rec-catR primers and the two genes were connected by an overlap extension PCR using the primers sacB-F and rec-catR (Table 2, Figure S1). One hundred ng of the PCR product and 10 ng of the linearized pET-27b by *Bam*H1 digestion were then co-transformed into the *E. coli* JM109 (DE3) cells harboring pKD46, and the positive clones were selected on kanamycin plates and the sequences of the resultant constructs were confirmed by DNA sequencing. The pRMT and pRMT-sacB plasmids were deposited in the Korean collection for type cultures: under KCTC 11994BP and KCTC 12478BP, respectively. Both materials will be available on request to the depositary or directly to the authors.

Next, the cloning cassette in the pRMT plasmid (T7 promoter - T7 terminator region) was grafted into pPROLar.A122 (Clontech, Mountain View, CA) and pCC1BAC (Epientre Biotechnologies) by PCR amplification and HR between the linear DNAs (Figure S1). The pRMT-p15A and pRMT-ori2 contained the cloning cassette at the location of the lac/ara-1 promoter in pPROLar.A122 and the lacZ gene in pCC1BAC-Km, respectively. Before the cloning cassette transfer, the *cat* gene of pCC1BAC was replaced with a kanamycin resistance gene.

Variants containing other reporter genes in the cloning cassette were also prepared by HR between the amplified PCR products of the pRMT plasmid and the streptomycin-resistance gene, the tetracycline-resistance gene, and the red fluorescent protein (RFP) gene from pCDFDuet-1 (Novagen), pACYC184 (New England Biolabs), and pTurboRFP (Evrogen), respectively (Figure S1).

### Preparation of target DNAs

To examine the effect of the homologous sequence length on the pRMT system, various primers with different length of homology arms were constructed such as 22/26, 32/36, 42/46, and 52/56 of forward_bp_/reverse_bp_. For cloning the inserts, we tested six linear DNA fragments, ranging from 0.7 to 10 kb. The DNAs were amplified using dual purpose primers for gene amplification and integration into the pRMT plasmids (Figures 1A and 1B, Figure S1). The forward primers consisted of the H1 region and the 5'-annealing sequence for the target gene, while the reverse primers contained the H2 region, the RBS plus ATG (22 bases), and the 3'-annealing sequence for the target gene. The primers used to prepare the insert are listed in Table 2. The gene encoding the green fluorescent protein (GFPuv, 0.7 kb) was amplified using the H1-GFPuvF and H2-GFPuvR primers. Two 2 kb genes, encoding the GFPuv-MBP and MBP-GFPuv fusion proteins, were prepared by overlap extension of the GFPuv gene and the maltose binding protein (MBP) gene in pMGFPuv [20]. 

For the preparation of the larger DNA fragments (4-10 kb), a gene cluster for the β-carotene synthesis pathway (4 kb) was amplified from *crtEBIY* (GenBank accession number: GQ149341) using the H1-crtE-F and H2-crtE-R primers (Table 2). The astaxanthin-producing gene cluster (6 kb) was prepared by adding 2 genes encoding the β-carotene ketolase from the *Erythrobacter* species (K143crtW: GenBank No. BAE47465) and the β-carotene hydroxylase from the *Paracoccus* species (A318crtZ: GenBank No. BAE47466) [21] to the 4 kb cluster using the primers listed in Table 2. To generate a 10 kb insert DNA, a 4 kb gene cluster containing mevalonate bottom pathway and *idi* gene was amplified from pSSN12Didi using the primers listed in Table 2, and connected with the above astaxanthin producing gene cluster (6 kb) by overlap extension PCR without primers for 13 cycles using KOD-FX (Toyobo, Osaka, Japan)[22]. The elongated DNA was then amplified for 30 cycles using KOD-FX. 

### *In vivo* cloning using pRMT vectors

The *E. coli* JM109 (DE3) cells with the receiver plasmid and the pKD46 plasmid were cultivated at 30°C in LB medium containing 10 μg/ml kanamycin, 100 μg/ml ampicillin, and 50 mM arabinose to induce the expression of the lambda-Red recombinase [10], and washed 2 times with ice-cold water and suspended in sterile 10% glycerol for the preparation of electro-competent cells (OD at 600 = 100). The electroporation was conducted with 100 ng of the PCR product in 50 μl of competent cells using a Gene Pulser Xcell System (Bio-Rad, Hercules, CA, USA). After electroporation, the cells were reactivated for 1 h at 37°C and the positive clones were selected on LB agar medium containing 25 μg/ml of chloramphenicol and validated by colony PCR using a T7 promoter and T7 terminator primers. 

The HR cloning using the pRMT-sacB plasmid was conducted using the same protocol except the positive colonies were selected on a medium containing 25 μg/ml of chloramphenicol and 7% sucrose for the counter-selection.

### Analysis

DNA concentrations were measured with a NanoDrop ND-1000 (Thermo Fisher Scientific, Wilmington, Delaware, USA). After homologous recombination, the colonies on plates were counted using a Gel Doc image analyzer (Bio-Rad, Hercules, CA, USA) and positive colonies were confirmed by colony PCR using the T7 promoter and T7 terminator primers. When the restriction pattern of the positive clone was analyzed, selected colonies were cultured in LB containing 25 μg/ml of chloramphenicol at 37°C. The plasmids were purified from the cells by an alkaline lysis method using Qiagen plasmid spin prep kit (Qiagen Inc., Valencia, CA, USA), and then digested with 5U of *Bgl*II (Takara Otsu, Shiga, Japan) and 5U of *Sca*I (Takara Otsu, Shiga, Japan). After gel electrophoresis, band intensities were analyzed using the Multi Gauge version 3.1 (Fuji Film, Tokyo, Japan) to calculate recombination yields ( plasmid fractions with inserts) of selected colonies.

## Results

### Rationale and characterization of the *in vivo* cloning system

**Figure 2:**
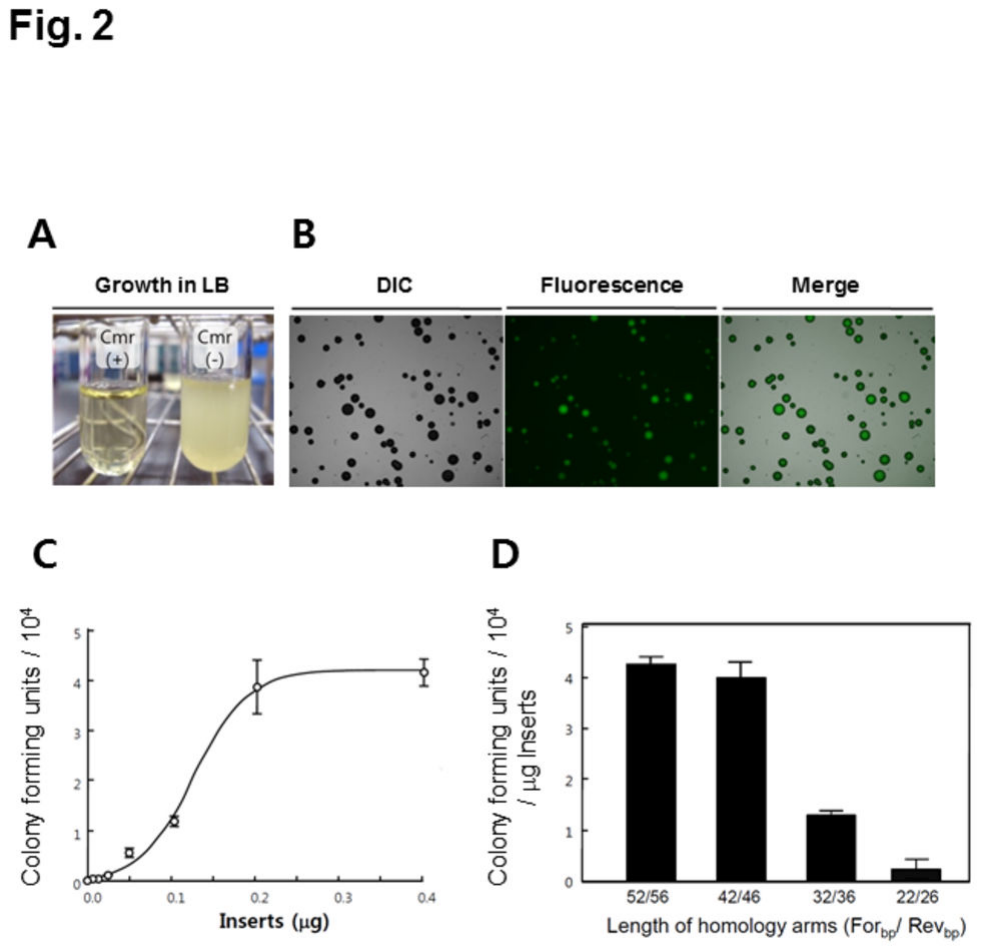
Restored antibiotic resistance and GFPuv expression by homologous recombination. (A) *E. coli* JM109 cells containing the pRMT plasmid were sensitive to 25 μg/l of chloramphenicol in LB broth because the chloramphenicol resistance gene is silent in the receiver plasmid. (B) GFPuv expression is observed in the positive colonies. (C) The effect of the amount of DNA insert on *in* *vivo* cloning. The DNA inserts were transformed into the cells containing the pRMT vector by electroporation. The colony number increases with higher concentrations of the insert used for electroporation. (D) The effect of the homologous sequence length on the pRMT system.

An RBS is an RNA sequence upstream of the start codon that is essential for initiating the translation of an open reading frame in prokaryotes. In this study, an RBS plus ATG sequence from PCR products was used as a switch to activate the translation of a silent reporter gene in receiver plasmids, enabling the direct cloning of the PCR products in living *E. coli* cells. The pRMT plasmid contain a specific cloning cassette between the T7 promoter (P_T7_)-terminator (T_T7_) region, as depicted in Figure 1A and 1B. The upstream homology region (H1) corresponds to the 42 nucleotides covering the *Xba*I and T7 RBS sites, while the downstream homology region (H2) corresponds to the 46 nucleotides covering the 5’-terminal sequence of the *cat* gene. The middle region between H1 and H2 consists of 36 bases, which excludes the RBS plus ATG codons (Figure 1). Therefore, the HR of an RBS plus ATG sequence enables the *E. coli* cells to grow in the presence of chloramphenicol, while the cells with only original plasmid show no detectable growth (Figure 2A). The *E. coli* cells co-transformed with the pRMT and pKD46 plasmids were preserved at -80°C and thawed immediately right before the use. 

The insert DNA was prepared to include the target gene and the 22 bases for the translation start in the middle of the H1 and H2 regions. When a GFPuv insert was tested as the DNA insert, the number of colonies on the antibiotic plates was 4.3 × 10^4^ for 1 μg of DNA and all of the colonies exhibited bright fluorescence as shown in Figure 2B, implying no significant false positives. The effect of DNA insert concentration on the number of colony forming units observed by changing the amount of GFPuv insert for electroporation from 0.005 to 0.4 μg. The amount of the pRMT vector in 50μl of electro-competent cells that have been used in this study was measured to be about 0.2 pmol. Thus the vector: insert DNA ratio for cloning was equimolar ratio when the 0.1 μg of GFPuv inserts (^≈^0.2 pmol) was used. The number of colonies was increased proportionally with the amount of DNA insert and reached saturation at 0.2 μg (≅0.4 pmol) (Figure 2C), which is similar with the previous report by Yu et al. [11].

Next, GFPuv genes were prepared by PCR amplification of the pRMT-GFPuv clones with various primers that resulted in different lengths in the homologous region and then transformed into *E. coli* JM109 (DE3) cells carrying the pRMT and pKD46 plasmids (Figure 2D). As a result, the colony forming units per µg of insert decreased drastically when shorter homology arms 32/36 and 22/26 were used (Figure 2D), further demonstrating that a shorter length for the homologous region lower than 40 bases is not appropriate for an HR reaction using the lambda Red HR system [6,7] . 

The stringent selection observed by inserting the RBS plus ATG sequence was also seen with other reporter genes, such as the tetracycline-resistance gene, the streptomycin- resistance gene, and the RFP gene, in the pRMT receiver plasmid (Figure S2). 

### Size dependency and parallel cloning of large gene clusters

**Figure 3:**
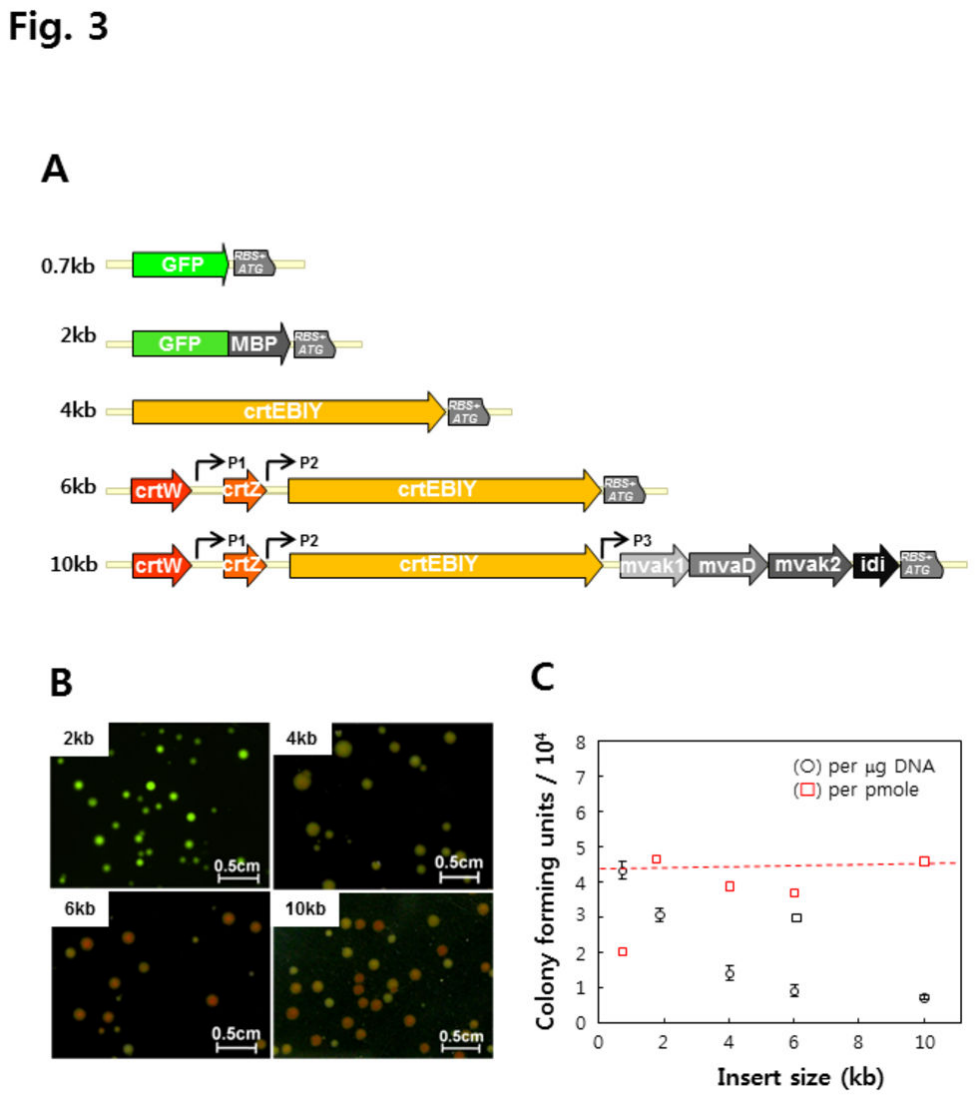
Size dependency and the cloning of the carotenoid synthetic gene clusters. (A) Shown are the genetic structures of the various carotenoid synthetic gene clusters used in this study. P_T7_ in synthetic gene cluster represent the T7 promoter. P1, P2, and P3 refer to additional promoters. (B) Shown are *E. coli* colonies expressing the various target genes: the 2-kb insert exhibited green fluorescence, while the 4-kb insert produced yellow-pigmented colonies. The 6-kb and 10-kb inserts showed mixed colonies of yellow and red, the colors of β-carotenoid and astaxanthin, respectively. (C) Analysis of the effect of insert size on the cloning efficiency.

The HR reaction is regarded as less dependent on the size of the DNA insert, making it more amenable for the cloning of large DNAs [7,8]. After validating the HR cloning system, we tested whether it was appropriate for the parallel cloning of large gene clusters, which is frequently required in protein and metabolic pathway engineering. Therefore, we generated 5 DNA inserts of different sizes; 2 inverted fusion constructs, GFPuv-MBP and MBP-GFPuv (2 kb), β-carotene production construct (4 kb), an astaxanthin synthesis construct (6 kb), and an astaxanthin-mevalonate pathway construct (10 kb) (Figure 3A). The carotenoid clusters were selected to take advantage of easy detection for protein expression based on the color-observation of the resulting colonies, which were β-carotene and astaxanthin for red and yellow, respectively. 

Each DNA insert was transformed into *E. coli* JM109 (DE3) cells along with the pRMT and pKD46 plasmids, and the resulting cells were then spread on a selection plate. As a result, a GFPuv-MBP clone (2 kb) showed 3.0 × 10^4^ colonies per μg of DNA and almost all were fluorescent green (Figures 3B and 3C). The inverse fusion, in which the MBP precedes the GFPuv, i.e., MBP-GFPuv, resulted in a 2.1-fold increase in colony numbers compared to the GFPuv-MBP; however the reason for this is unclear. The number of positive colonies with the carotenoid gene clusters (4 kb) was 1.4 × 10^4^ colonies per μg DNA and the majority of the colonies were yellow, which is the color of β-carotene (Figures 3B and 3C). The 6 kb-astaxanthin synthesis cluster included additional oxygenation reactions from β-carotene, which are catalyzed by the products of *crtW* and *crtZ* [21]. As a result, the number of positive clones was 0.91 × 10^4^ colonies per μg of DNA and almost all of the colonies were red or yellow, representing β-carotene or astaxanthin, respectively (Figure 3B and 3C). The final test was with the 10 kb insert containing multiple promoters and genes that encode astaxanthin via the mevalonate pathway [21,23], as depicted in Figure 3A. This large DNA cluster was comprised *crtEBIY*, K143*crtW*, and A318*crtZ*, which produce astaxanthin (6 kb), and *mvak1, mvakD*, and *mvak2*, which constitute the mevalonate bottom pathway genes (3 kb) from *Streptococcus pneumoniae* and *idi* which encodes isopentenyl diphosphate isomerase (0.55 kb) from *E. coli* [24,25]. When the 10 kb insert was tested on the selection plates with 20 μg/ml mevalonate, the colony number was 0.72 × 10^4^ colonies per μg of PCR product and the color of the colonies was red or yellow (Figures 3B and 3C). Although the colony numbers were low while using larger inserts (circles in Figure 3C), the calculations based on the molecular masses of the DNA inserts revealed that the cloning efficiencies of the large carotenoid clusters were comparable with those of the smaller DNA inserts (red squares in Figure 3C). Therefore, the HR cloning in this study is estimated to be useful for the parallel cloning of large DNAs carrying multiple genes and promoters.

### Improvement of plasmid homogeneity in transformant

**Figure 4:**
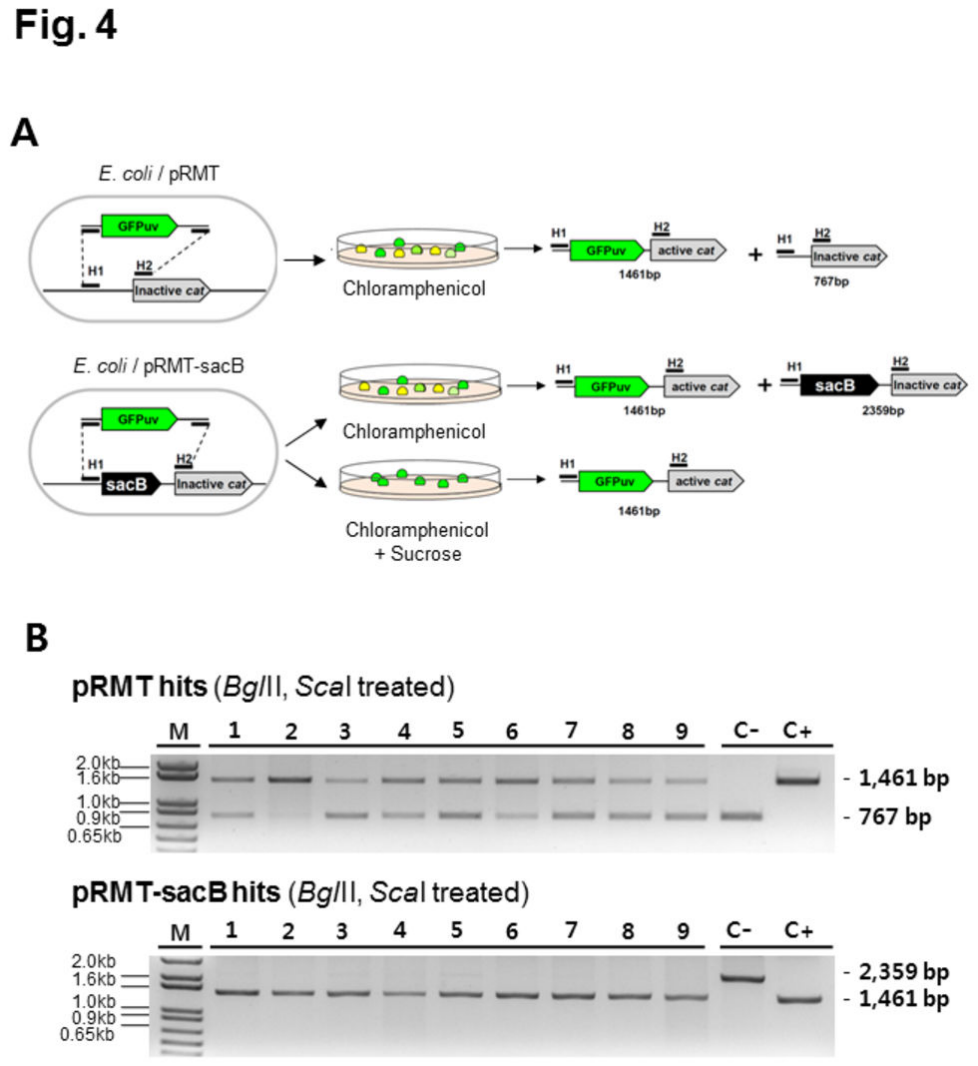
The homogeneity problem and the solution using a counter selection marker, *sacB*. (A) Shown are the genetic structure and scheme of the pRMT and pRMT-sacB plasmids when the GFPuv gene was recombined into both plasmids. (B) Shown is the restriction analysis of the target plasmids from selected hits. upper and lower panels represent the electrophoresis results from the pRMT and pRMT-sacB plasmids, respectively, digested with *Bgl*II and *Sca*I. The C- and C+ represent the indicator bands of one without and one with the insert, respectively while the GFPuv gene was used as a target gene.

Despite the high resistance to antibiotics, the selected cells contained a mixture of recombinant and parental plasmids with or without the insert DNA (Figure 4A), because the pRMT plasmid is present in large copy number in the cell. Thus, the cells exhibited two DNA bands of different intensities on PCR analyses using the T7 promoter and terminator primers (Figure S3). Unfortunately, increasing the concentration of chloramphenicol on the selection plates did not significantly reduce the number of plasmids without an insert (data not shown).

In an effort to improve the homogeneity of the colonies, we cloned the H1-H2 region into pPROLar.A122 and pCC1BAC with the lower copy origin p15A and the single copy origin ori2, respectively. Additionally, the H1-H2 region was cloned into the *E. coli* JM109 (DE3) chromosome at the location of the *bglA* gene, following the general protocols using pKD46 [10]. The GFPuv gene with 42/46 homology arms was transformed into the cells as the insert. However, with the pRMT- p15A plasmid, the non-homogeneity of the recovered plasmids was not improved while the colony numbers were slightly decreased (Figure S4). Moreover, the pRMT-ori2 plasmid and chromosomal integration system resulted in a significant decrease in the number of positive colonies, resulting in 0.01 × 10^4^ colonies per μg of DNA, which corresponds to approximately 1% of the level from the pRMT-based method (Figure S4). 

Next, to remove the parental plasmid in the positive cells; this study introduced a counter selection marker, *sacB*, which encodes a levansucrase that converts sucrose to a harmful levan in *E. coli* cells (Figure 4A). The s*acB* gene was inserted between the H1 and H2 region, substituting the middle region constituting of 36 base (Figure 1). Transformation with the pRMT-sacB plasmid resulted in 0.41 × 10^4^ positive colonies per μg of the GFPuv genes in the presence of 25 μg/ml of chloramphenicol and 7% sucrose, which was a 9.5% of that when using the plasmid without *sacB*. 

To validate the improved homogeneity, 9 randomly selected colonies from the pRMT and pRMT-sacB transformations were transferred to the LB broth containing 25 μg/ml of chloramphenicol and grown at 37°C. The plasmids were purified and subsequently digested with *Bgl*II and *Sca*I (Figure 4B). The band thickness in electrophoretic analysis showed that the pRMT plasmid without an insert constituted between 28-72% of the total purified plasmids in the randomly selected colonies (the upper panel of Figure 4B). However, the same analysis detected no other bands when using the pRMT-sacB plasmid (Figure 4B), verifying that the present method yielded homogeneous clones in *E. coli* cells, by a simple transformation of PCR products.

## Discussion

Gene cloning and DNA manipulation based on HR have lately attracted much attention owing to their convenient advantages in not requiring a restriction enzyme digestions and ligation reaction [1] thus it gives more freedom for cloning [6,7]. This makes HR cloning a good choice to meet the increasing demands for high throughput and parallel gene cloning of synthetic gene clusters [1,4,26]. The plasmid vectors, however, need to be linearized completely before their application to minimize the false positive clones [6,8], otherwise the DNA insert has to include a full-size selection marker in the construct [10]. Nonetheless, this study presented a new method to control the selection marker expression by switching of a short RBS plus ATG sequence from the DNA insert to the receiver plasmid (Figure 1). The resulting screen for positive hits was stringent and the cells without the insert showed no proliferation in the presence of chloramphenicol (Figure 2A), streptomycin, and tetracycline (Figure S2), enabling robust and rapid screening on solid plates. A similar attempt to control the expression of reporters with a truncated promoter was previously attempted. However, the screening was limited by the leaky activity of the truncated promoter [27]. 

Our restriction analysis of the plasmids in the selected colonies exhibited two bands one with and one without the insert because some of the parental plasmid remained intact in the positive cells (upper panel in Figure 4B). Increasing the amounts of antibiotics in the selection media did not eliminate the intact plasmid, probably because the low level of HR may fulfill the required resistance to antibiotics in cells. The pRMT plasmid (a pBR322 origin) is known to be present in 15~20 copies in *E. coli* cells and the yields of recombination in cells can be changed by the availability of insert DNAs in cells. In this study, the 0.1 µg of GFPuv (size: 0.7 kb) corresponds to 0.2 pmol that is equimolar ratio with pRMT plasmids in 50 µl of electroporation solution. Inside the competent cell however, the ratio could be even lower. Therefore, the broad recombination yield of 28-72% is estimated to be related with the lower insert DNA ratio in the cell during transformation. Unfortunately, using the single copy number plasmid, pRMT-ori2, or cloning into the *E. coli* JM109 chromosome at the location of the *bglA* gene resulted in a significant decrease in the number of positive colonies (Figure S4). 

Finally, introduction of the *sacB* into the receiver plasmid as a counter selection marker was successful in overcoming the homogeneity problem, yielding a number of positive colonies with the proper insert on agar plates. 

## Conclusions

The demand for cloning and expression of large gene clusters is increasing [26]. This study presents a simple and rapid method for *in vivo* cloning that is useful for the cloning and expression of large DNA carrying multiple genes, requiring only the transformation of linear DNAs into *E. coli* cells. The switching of an RBS plus ATG sequence by HR resulted in completely homogeneous clones in living cells when combined with counter selection using the *sacB* gene. In addition, the high efficiency of this method is useful for parallel gene cloning and rapid preparation of genetically diverse libraries in *E. coli*. 

## Supporting Information

Figure S1 **Schematic representation for construction of pRMT and pRMT-sacB vectors as receiver plasmids.** The backbone of pRMT vectors was taken from pET27b and all insert DNAs were prepared by PCR of different templates. Then, two linear DNAs were electro-transformed into *E. coli* cells containing pKD46 plasmid for homologous recombination.
(TIF)

Figure S2 **Stringent selection by switching of ribosome binding sequence in different reporter proteins in pRMT vector.** (A) Cell growth of *E. coli* pRMT-reporter variants in LB broth containing 25 μg/L of chloramphenicol, 10 μg/L of tetracycline, and 10 μg/L of streptomycin, respectively. (B) Fluorescence imaging of *E. coli* pRMT with RFP as the reporter protein. ‘N’ indicates no RBS plus ATG is included in the upstream sequence of the reporter gene.
(TIF)

Figure S3 **Non-homogeneity problem of pRMT vectors in transformant.** PCR analyses of GFPuv-producing colonies with T7 promoter and T7 terminator primers. C+ and C- indicates the positive control with insert and the negative control without insert, respectively.
(TIF)

Figure S4 **Effect of plasmid copies on the cloning efficiency of GFPuv insert by homologous recombination in pRMT system.** The cloning cassette was transferred into pBR322, pPROLar, and pCC1BAC plasmids with pMB1, p15A, and Ori2 origin, respectively. Also, the cassette was also transferred into the *E. coli* JM109 (DE3) chromosome at the location of the *bglA* gene, following the general protocols using pKD46.
(TIF)
