# Supplementary figures and images for: Generating In Vivo Cloning Vectors for Parallel Cloning of Large Gene Clusters by Homologous Recombination
Source: PLoS One. 2013 Nov 11;8(11):e79979. doi: 10.1371/journal.pone.0079979 (PMC3823602; doi:10.1371/journal.pone.0079979)

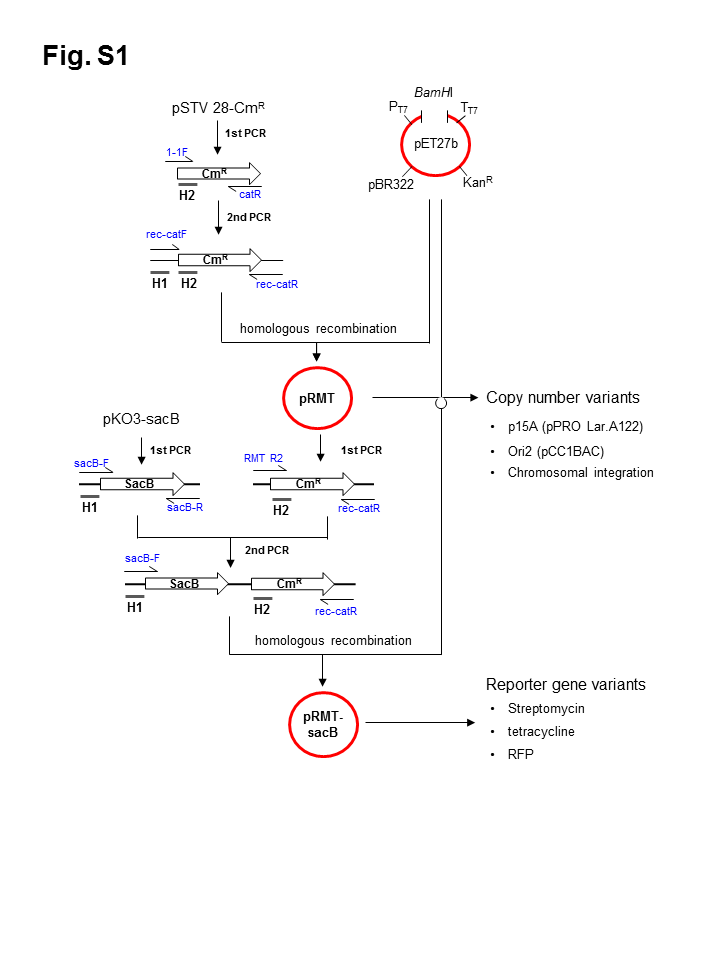

Supplement: Figure S1 — Schematic representation for construction of pRMT and pRMT-sacB vectors as receiver plasmids. The backbone of pRMT vectors was taken from pET27b and all insert DNAs were prepared by PCR of different templates. Then, two linear DNAs were electro-transformed into E. coli cells containing pKD46 plasmid for homologous recombination. (TIF) [file pone.0079979.s001.tif]
